# A Case of Spontaneous Evolution of the Fœtus

**Published:** 1791

**Authors:** Richard Simmons

**Affiliations:** one of the Surgeons of the British Lying-in Hospital in London.


VI. *A Caſe of ſpontaneous Evolution of the Fœtus. Communicated in a Letter to Dr.* Simmons, *F. R. S. by Mr.* Richard Simmons, *one of the Surgeons of the Britiſh Lying-in Hoſpital in London.*

EVERY one who is much engaged in the practice of midwifery muſt have experienced the difficulty, and even danger, with which the operation of turning a child is ſometimes attended; and in caſes where the arm preſents, and from the ſtrong action of the uterus, it may be hazardous, or even impoſſible to deliver by the feet, it muſt be a pleaſing circumſtance to know that nature, unaſſiſted, is capable of effecting the delivery by bringing about a ſpontaneous evolution of the fœtus.

This curious and valuable fact, for which we are indebted to Dr. Denman*, is more particularly applicable to thoſe caſes of arm preſenta-

* See London Medical Journal, Vol. V. pages 64 and 301.

tion tion where the child is known to be dead; because in ſuch caſes our ſole object muſt be to bring away the fœtus in ſuch a way as ſhall be the eaſieſt and ſafeſt for the mother; and we ſhall be more diſpoſed to wait with patience for the ſpontaneous operations of nature, when we are certain there is no longer any proſpect of ſaving the life of the child.

The number of inſtances of this ſpontaneous evolution, hitherto publiſhed, being ſmall, and there appearing to be no inconſiderable variety, both as to the length of time and the manner in which the evolution takes place, I am induced to communicate to you the following ſhort account of a caſe which occurred to me lately in the Britiſh Lying-in Hoſpital, and which fully corroborates the ingenious obſervations of Dr. Denman on this ſubject.

Ann Collins, thirty-ſix years of age, and of a middle ſtature, was taken in labour of her ſixth child about ten o'clock on Tueſday evening, December the 21ſt, 1790. She had ſlight pains till about two o'clock the following morning. The labour pains began then to grow ſtronger, and continued to do ſo till about four o'clock; when the midwife, upon examination, found that the left arm of the child had made its way into the vagina. I was now ſent for, and by the time time I reached the patient the arm had advanced as low down as the shoulder, and was hanging entirely out of the os externum.

The midwife being unable to give me any account at what time the membranes had broken, I was desirous, during the interval of the pains, of introducing my hand into the uterus, in order to effect the delivery by turning; but this, from the frequency and violence of the pains, I found impracticable, without using more force than I was then willing to do.

In a short time the left breast of the child was forced out of the os externum; and as in this case nature seemed likely to do so much, I was now in expectation that a spontaneous evolution of the fœtus would take place: and in this I was not deceived, for in about half an hour from the time I came to the patient, and not more than two hours from the first appearance of the arm in the vagina, the child was completely turned by the mere action of the uterus, and was expelled as if the breech had originally presented. The child was rather a large one; the placenta came away very properly; and the woman recovered without any thing particular happening to her.

*Newman Street,*
March 27, 1791.

